# Preoperative identification and biopsy of sentinel lymph nodes using contrast-enhanced ultrasound: the Tunbridge Wells experience

**DOI:** 10.1186/bcr3767

**Published:** 2015-11-05

**Authors:** Tania De'Silva, Mohamed Hashem, Nick Wakeham, Sarah Kirwan, Neal Chayya, Pippa Mills, Karina Cox

**Affiliations:** 1 Maidstone and Tunbridge Wells NHS Trust, Tunbridge Wells, UK

## Introduction

At Maidstone and Tunbridge Wells NHS Trust, all newly diagnosed breast cancer patients with a normal grey-scale axillary ultrasound have a procedure to identify and biopsy sentinel lymph nodes (SLN) using contrast-enhanced ultrasound (CEUS).

## Methods

Retrospective data were collected on patients undergoing a CEUS guided biopsy over a 42-month period at Tunbridge Wells Breast Clinic (TWBC). We compared the results of the first group of patients with the most recent to determine the performance of the test over time.

## Results

Between February 2011 and June 2012, 94 patients had a CEUS guided biopsy of SLN. Twenty patients were excluded; five had neoadjuvant therapy, five were unfit for surgery, one had an abnormal grey-scale ultrasound and nine had incomplete data. SLN were visualised in 92% and 83% had a successful SLN core biopsy. The sensitivity of the test to detect SLN metastases was 56%, specificity 100%, negative predictive value 86% and prevalence of lymph node (LN) metastases 27%. Between October 2013 and September 2014, 99 patients had the test. Thirty patients were excluded from the final analysis. SLN were visualised in 86% and 74% had a successful SLN core biopsy. The sensitivity was 69%, specificity 100%, negative predictive value 90% and prevalence of LN metastases 25%.

## Conclusion

The percentage of SLN visualised and successfully biopsied in TWBC decreased between the two study periods. These findings may represent a normal fluctuation of the test's performance, be a function of missing data in retrospective collection or the cumulative 'learning-curves' of newly appointed radiologists. This warrants further analysis.

